# Acute kidney injury as a key predictor of cardiovascular events in chronic kidney disease patients: the CKD-REIN study

**DOI:** 10.1093/ckj/sfae337

**Published:** 2024-12-11

**Authors:** Nans Florens, Estelle Aymes, Victoria Gauthier, Luc Frimat, Maurice Laville, Dimitri Bedo, Thomas Beaudrey, Philippe Amouyel, Nicolas Mansencal, Céline Lange, Sophie Liabeuf, Ziad A Massy, Benedicte Stengel, Natalia Alencar de Pinho, Aghiles Hamroun

**Affiliations:** Nephrology, Dialysis & Transplantation Department, Nouvel Hôpital Civil, Hôpitaux Universitaires de Strasbourg, 1 Place de l'hôpital, Strasbourg, France; UMR1109 Molecular Immuno-Rhumatology, FHU TARGET, Translational Medicine Federation of Strasbourg (FMTS), Faculty of Medicine, University of Strasbourg, Strasbourg, France; INI-CRCT (Cardiovascular and Renal Trialists), F-CRIN Network, Vandoeuvre-les-Nancy, France; Public Health Department, Epidémiologie – Maison régionale de la recherche clinique, CHU Lille, Lille, France; UMR1167 RIDAGE, Institut Pasteur de Lille, INSERM, Univ Lille, Lille, France; Public Health Department, Epidémiologie – Maison régionale de la recherche clinique, CHU Lille, Lille, France; UMR1167 RIDAGE, Institut Pasteur de Lille, INSERM, Univ Lille, Lille, France; Nephrology Department, CHRU de Nancy, Vandoeuvre-lès-Nancy; Lorraine University, APEMAC, Vandoeuvre-lès-Nancy, France; Université de Lyon, Carmen INSERM, Lyon; Nephrology, Dialysis & Transplantation Department, Nouvel Hôpital Civil, Hôpitaux Universitaires de Strasbourg, 1 Place de l'hôpital, Strasbourg, France; UMR1109 Molecular Immuno-Rhumatology, FHU TARGET, Translational Medicine Federation of Strasbourg (FMTS), Faculty of Medicine, University of Strasbourg, Strasbourg, France; Nephrology, Dialysis & Transplantation Department, Nouvel Hôpital Civil, Hôpitaux Universitaires de Strasbourg, 1 Place de l'hôpital, Strasbourg, France; UMR1109 Molecular Immuno-Rhumatology, FHU TARGET, Translational Medicine Federation of Strasbourg (FMTS), Faculty of Medicine, University of Strasbourg, Strasbourg, France; Public Health Department, Epidémiologie – Maison régionale de la recherche clinique, CHU Lille, Lille, France; UMR1167 RIDAGE, Institut Pasteur de Lille, INSERM, Univ Lille, Lille, France; Cardiology Department, Centre de référence des cardiomyopathies et des troubles du rythme cardiaque héréditaires ou rares, AP-HP, Ambroise Paré Hospital, Université de Versailles-Saint Quentin (UVSQ), Boulogne-Billancourt, France; Centre for Research in Epidemiology and Population Health (CESP), Paris-Saclay University, INSERM U1018, Versailles-Saint-Quentin University, Clinical Epidemiology Team, Villejuif, France; Centre for Research in Epidemiology and Population Health (CESP), Paris-Saclay University, INSERM U1018, Versailles-Saint-Quentin University, Clinical Epidemiology Team, Villejuif, France; Agence de la Biomédecine, La Plaine Saint-Denis, France; Pharmacoepidemiology Unit, Department of Clinical Pharmacology, Amiens-Picardie University Medical Center, MP3CV Laboratory, Jules Verne University of Picardie, Amiens, France; INI-CRCT (Cardiovascular and Renal Trialists), F-CRIN Network, Vandoeuvre-les-Nancy, France; Centre for Research in Epidemiology and Population Health (CESP), Paris-Saclay University, INSERM U1018, Versailles-Saint-Quentin University, Clinical Epidemiology Team, Villejuif, France; AURA Paris - Association pour l'Utilisation du Rein Artificiel en région Parisienne, and Department of Nephrology, Ambroise Paré University Hospital, APHP, Boulogne-Billancourt, Paris, France; Centre for Research in Epidemiology and Population Health (CESP), Paris-Saclay University, INSERM U1018, Versailles-Saint-Quentin University, Clinical Epidemiology Team, Villejuif, France; Centre for Research in Epidemiology and Population Health (CESP), Paris-Saclay University, INSERM U1018, Versailles-Saint-Quentin University, Clinical Epidemiology Team, Villejuif, France; Public Health Department, Epidémiologie – Maison régionale de la recherche clinique, CHU Lille, Lille, France; UMR1167 RIDAGE, Institut Pasteur de Lille, INSERM, Univ Lille, Lille, France

**Keywords:** acute kidney injury, cardiovascular events, chronic kidney disease, CKD-REIN, risk factors

## Abstract

**Background and Hypothesis:**

Cardiovascular diseases are a leading cause of morbidity and mortality in patients with chronic kidney disease (CKD). Acute kidney injury (AKI) has been increasingly recognized as a potential exacerbating factor for cardiovascular events in these patients. The CKD-REIN study aims to explore the relationship between AKI and the risk of major adverse cardiovascular events (MACE) in a cohort of CKD patients. We hypothesize that AKI is a significant and independent predictor of MACE in patients with CKD, and that the severity of AKI correlates with the risk of subsequent cardiovascular events.

**Methods:**

This prospective cohort study included 3033 adult CKD patients from 40 outpatient nephrology clinics in France. Patients were followed for a median of 5.2 years. AKI episodes were identified and staged based on the KDIGO-AKI criteria. Cardiovascular events, including myocardial infarction, stroke, heart failure hospitalization, and cardiovascular death, were systematically recorded. The association between AKI and MACE was analyzed using a multivariable Cox model, adjusting for confounders such as demographic characteristics, medical history, and baseline kidney function.

**Results:**

During the follow-up, 530 patients experienced at least one episode of AKI. The cumulative incidence of MACE at 1 year post-AKI was 8.1%. Patients with AKI had a significantly increased risk of MACE, with an adjusted hazard ratio (HR) of 5.78 (*P* < .001). The risk was consistent across different MACE components and was independent of age, sex, CKD stage, or comorbidities. The risk of MACE was higher for more severe AKI stages and for AKI events requiring hospitalization or associated with incomplete renal recovery.

**Conclusion:**

The findings of this study confirm that AKI is a significant independent predictor of MACE in CKD patients, demonstrating a strong severity–response relationship. These results underscore the importance of vigilant cardiovascular monitoring and preventive strategies in CKD patients following AKI episodes. Understanding the mechanisms linking AKI to cardiovascular outcomes is crucial for developing targeted interventions to mitigate these risks.

## Graphical Abstract

**Graphical Abstract:**
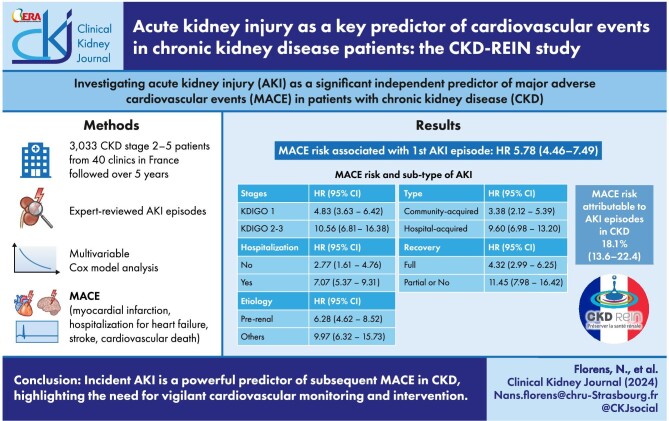

KEY LEARNING POINTS **What was known:**

- Acute kidney injury (AKI) has been associated with increased cardiovascular risk, but its role as an independent predictor of major adverse cardiovascular events (MACE) in chronic kidney disease (CKD) patients was not fully established.

**This study adds:**

- This study demonstrates that AKI is a significant independent predictor of MACE in CKD patients, with a strong severity–response relationship based on AKI severity.

**Potential impact:**

- The findings highlight the need for enhanced cardiovascular monitoring and preventative strategies in CKD patients following AKI episodes, potentially improving patient outcomes and reducing cardiovascular events.

## INTRODUCTION

Cardiovascular mortality and morbidity represent significant health challenges in contemporary society [1]. Chronic kidney disease (CKD) is identified as a crucial, non-traditional cardiovascular risk factor [2]. A robust association exists between reduced glomerular filtration rate (GFR), elevated albuminuria and the increased incidence of cardiovascular events [3–5]. Furthermore, recent findings from an extensive Mendelian randomization study have associated mild to moderate CKD with a heightened incidence of cardiovascular events [6], suggesting a pathophysiological interconnection between the heart and kidneys [7], which is also corroborated by experimental studies [8–10].

When considering acute kidney injury (AKI) events, only a limited number of studies have established a link with long-term cardiovascular outcomes [11–14]. In the meta-analysis by Odutayo *et al*., AKI related to heightened risks of major adverse cardiovascular events (MACE), such as acute myocardial infarction, stroke, heart failure, and cardiovascular death [13]. However, as recognized by the authors, these conclusions are limited by the diverse definitions of AKI used, the variability in follow-up periods, the restricted set of confounding factors examined, and the focus on highly selected populations, particularly in scenarios involving ischemia-reperfusion injury. Currently, the potential association between AKI and MACE within the CKD population is even less well explored [15]. Given the shared cardiovascular risk factors between AKI and CKD, the relationship between kidney diseases (acute or chronic) and the risk of subsequent MACE continues to be contentious [12, 14].

We hypothesize that AKI is a significant and independent predictor of cardiovascular events in the CKD population. Using data of a prospective national cohort study of patients with moderate-to-severe CKD, we here investigate the connection between incident AKI episodes and the subsequent occurrence of MACE, including acute myocardial infarction, stroke, hospitalization for heart failure, and cardiovascular death.

## MATERIALS AND METHODS

The results of this cohort study are reported in accordance with the Strengthening the Reporting of Observational Studies in Epidemiology (STROBE) guidelines [16].

### Study design and participants

CKD-REIN is a prospective cohort study conducted in 40 nationally representative outpatient nephrology centers in France. Eligible adult patients had a confirmed diagnosis of stage 2–5 CKD based on two values of estimated GFR (eGFR) at 3-month intervals during the screening period; they were not on dialysis and had not undergone kidney transplantation. Between July 2013 and March 2016, the CKD-REIN investigators enrolled 3033 patients, all of whom gave their written, informed consent. Details of the study protocol and study flow chart have been published previously [17, 18]. The study was approved by the institutional review board at the French National Institute of Health and Medical Research (INSERM; reference IRB00003888) and was registered at ClinicalTrials.gov (NCT03381950).

### Study data

Data were collected at baseline and then annually by trained clinical research associates (CRAs) from patient interviews and medical records. The study data included baseline socio-demographic characteristics and medical background including any history of diabetes, obesity, cirrhosis, cancer, low birth weight, dyslipidemia, tobacco use, cardiovascular disease, or AKI. Participants underwent blood and urine tests, including measurements of serum creatinine, hemoglobin, uric acid, albumin, and urinary albumin-to-creatinine ratio. The 2009 creatinine-based Chronic Kidney Disease Epidemiology Collaboration equation was used to estimate the baseline GFR [19]. Additionally, to account for the decline in kidney function, the individual slope of GFR decline was estimated prior to inclusion for all patients through mixed effects modeling [11]. This estimation was derived from an average of five values per patient, spread over 1.2 years before inclusion.

Patients were asked to bring all their drug prescriptions for the preceding 3 months (for the enrollment visit) or all the year's prescriptions (for each annual follow-up visit). Accordingly, drug prescriptions were continuously recorded from 3 months preceding study inclusion to the end of the follow-up period. We used the Anatomical Therapeutic and Chemical thesaurus to code medications, and we recorded the drug start and stop dates [20]. Medication adherence was assessed with the validated, questionnaire-based Girerd score [21]. Polypharmacy was defined as the prescription of >10 different therapeutic classes per day. Longitudinal data (appointments with the nephrologist, hospital admissions, laboratory tests: whether prescribed annually as per the study protocol or routinely by the nephrologist or by any other physician) were recorded at 1-year intervals.

### Exposure definition: identification and staging of AKI events

During the study follow-up, all AKI episodes in outpatients or inpatients were identified. Several sources were used to identify AKI episodes: (i) the study's CRAs were trained to identify any mention of AKI in hospital reports, and (ii) a study physician reviewed all the hospital discharge reports for any mention of elevated serum creatinine levels, regardless of whether AKI was specified. Additional data on the AKI adjudication process in the CKD-REIN cohort have been published elsewhere [22]. The AKI classification was based on the 2012 KDIGO-AKI criteria, using the baseline and peak serum creatinine levels as follows: Stage 1: 1.5–1.9 × baseline serum creatinine or ≥0.3 mg/dl increase in serum creatinine; Stage 2: 2.0–2.9 × baseline serum creatinine; Stage 3: 3 × baseline serum creatinine or ≥4.0 mg/dl increase, or dialysis initiation [23]. The baseline serum creatinine was set as the average of the values measured in the year preceding the AKI [24]. Hospital-acquired AKI refers to AKI that occurred during a hospital stay, whereas community-acquired AKI refers to events that did not involve a hospitalization or were reported as a cause of hospital admission. Only the occurrence of the first incident episode of AKI during the follow-up period was used to define exposure, considering its time-dependent nature.

### Outcome definitions

Hospitalizations occurring during study follow-up were identified from medical reports, hospital records, and/or patient interviews; deaths were ascertained from death certificates, hospital records, reports by family members, and by linkage with the French national death registry [25]. A physician reviewed and coded all events using the International Classification of Diseases version 10 (ICD-10). In addition, this physician classified all cardiovascular events according to the Cardiovascular and Stroke Endpoint Definitions for Clinical Trials and a cardiologist adjudicated all CV deaths [26]. The definition of a MACE thus encompasses the occurrence of one of the following events: acute myocardial infarction, stroke, hospitalization for heart failure, or cardiovascular-related death.

Kidney failure with replacement therapy (KFRT) events were defined as initiation of maintenance dialysis or pre-emptive kidney transplantation, identified from medical records, patient interviews, or by linkage with the national REIN (Renal Epidemiology and Information Network) registry [27].

### Statistical analyses

Baseline characteristics were described for the participants as a whole and according to a previous history of cardiovascular events before inclusion. Data were expressed as the median [Q1, Q3], or the number (percentage).

For the primary analysis examining the relationship between AKI and MACE, observations were followed from cohort inclusion until the first event among MACE, kidney failure (dialysis or transplantation), non-cardiovascular death, or the date of last follow-up, whichever occurred first. Cumulative incidences of MACE after AKI events were estimated using the Aalen–Johansen method, considering competitive risks of kidney failure and non-cardiovascular death. These cumulative incidences were compared according to the in-/outpatient and community-/hospital-acquired status of the AKI event, using the Gray method. The relation of incident AKI on the risk of subsequent MACE was assessed using a multivariable cause-specific Cox model, with AKI modeled as a time-dependent variable and stratified according to various characteristics (AKI stage, in-/outpatient, pre-renal etiology, hospital-/community-acquired, and renal recovery at hospital discharge). Results were expressed as hazard ratios and 95% confidence intervals. Adjustment variables were selected *a priori* based on their clinical relevance and a literature review, and the final set was retrieved using a directed acyclic graph [28]. In consequence, all the multivariable analyses included: socio-demographic characteristics (age, sex, education, tobacco use), personal medical history (hypertension, coronary artery disease, cerebrovascular disease, heart failure, peripheral artery disease, diabetes, obesity, dyslipidemia, cirrhosis, cancer, low birth weight), history of past-AKI before inclusion, markers of CKD severity (eGFR at baseline and estimated annual slope before inclusion, urinary albumin-to-creatinine ratio), other laboratory data (serum hemoglobin, uric acid and albumin levels at baseline), and drug prescriptions [proton pump inhibitors, diuretics, beta-blockers, anti-inflammatory drugs, renin-angiotensin-aldosterone system (RAAS) inhibitors, polypharmacy, and treatment adherence]. Considering the expected risk of discontinuation of RAAS inhibitors associated with the primary exposure (AKI), their prescription was also treated as a time-dependent variable in the models [29]. Secondary analyses were conducted to examine the association between AKI and each of the events involved in the definition of MACE, individually. The fraction of MACE risk attributable to AKI events (and its 95% confidence interval) was derived using the effect size obtained from the primary model.

We investigated the potential modifying effect of sex, age (<75 versus ≥75 years), diabetes, CKD stage (stage 2–3 versus 4–5), baseline history of AKI, or cardiovascular disease by testing interactions between these characteristics and AKI in the association with MACE. Since some patients experienced an AKI and MACE event concurrently (i.e. within the same 24 hours), a sensitivity analysis was also conducted by censoring these observations, assuming that AKI occurred after MACE. Finally, the primary analysis was also conducted after exclusion of patients with a history of AKI and/or cardiovascular event(s) before inclusion.

The proportional hazards assumption was checked with the Schoenfeld residuals test. Continuous variables were included in the different models without transformation after we verified the log-linearity assumption. Missing data were managed by multiple imputation with chained equations (20 iterations, 20 datasets), including all covariates in the Cox models. We used Rubin's rules to combine estimates from each regression model across the imputed datasets [30].

The significance threshold for all statistical tests was set at 5%. The analyses were performed using R software, version 3.6.2.

## RESULTS

### Baseline characteristics

Among the 3033 patients included in the cohort (median age of 69 years, 65.4% male, median eGFR of 32.8 ml/min/1.73 m²), 758 of them (25%) had a cardiovascular history at baseline, corresponding to a prevalence of coronary artery disease, stroke, or heart failure of 24.8%, 10.0%, and 13.0%, respectively (Table 1). Patients with a history of major cardiovascular events were significantly older, more often male, had lower education levels, and more cardiovascular risk factors (diabetes, obesity, hypertension, and smoking). It is noteworthy that they also more frequently had a history of AKI and markers of poorer kidney function (lower eGFR, higher albuminuria levels). Finally, these patients were also more often treated with diuretics, beta-blockers, lipid-lowering agents, and proton pump inhibitors.

**Table 1:** Characteristics of the study population, according to a history of major cardiovascular event (i.e. myocardial infarction, stroke, or heart failure) at baseline.

		History of major cardiovascular event			
	All	No	Yes		Missing data
	(*N* = 3033)	(*N* = 2275)	(*N* = 758)	*P* value	(%)
**Socio-demographic characteristics**					
Age (years)	69.0 [60.0,76.0]	67.0 [58.0,75.0]	72.0 [66.0,78.0]	<.001	
Male sex	1983 (65.4%)	1405 (61.8%)	578 (76.3%)	<.001	
Education					
<9 years	443 (14.8%)	314 (13.9%)	129 (17.4%)	.002	37 (1.2%)
9–11 years	1471 (49.1%)	1088 (48.3%)	383 (51.5%)		
≥12 years	1082 (36.1%)	851 (37.8%)	231 (31.1%)		
Occupational status					
Unemployed or retired	2199 (82.9%)	1599 (79.5%)	600 (93.5%)	<.001	380 (12.5%)
Employed	454 (17.1%)	412 (20.5%)	42 (6.5%)		
**Kidney disease history**					
CKD vintage (years)	5.11 [2.45,10.0]	5.41 [2.56,10.8]	4.52 [2.30,8.17]	<.001	142 (4.7%)
Primary kidney disease					
Diabetic nephropathy	611 (21.4%)	404 (18.9%)	207 (29.1%)	<.001	181 (6.0%)
Hypertensive/vascular nephropathy	849 (29.8%)	566 (26.4%)	283 (39.7%)		
Other	1392 (48.8%)	1170 (54.7%)	222 (31.2%)		
AKI history	658 (23.6%)	452 (21.7%)	206 (29.2%)	<.001	244 (8.0%)
CKD stage					
2–3	1725 (56.9%)	1332 (58.5%)	393 (51.8%)	.001	.
4–5	1308 (43.1%)	943 (41.5%)	365 (48.2%)		
**Personal medical history**					
Heart failure	392 (13.0%)	28 (1.2%)	364 (48.0%)	<.001	8 (0.3%)
Coronary artery disease	737 (24.8%)	242 (10.9%)	495 (65.6%)	<.001	63 (2.1%)
Cardiac rhythm disorder	674 (22.3%)	374 (16.5%)	300 (39.6%)	<.001	8 (0.3%)
Stroke	297 (10.0%)	61 (2.8%)	236 (31.4%)	<.001	75 (2.5%)
Peripheral artery disease	574 (19.4%)	316 (14.3%)	258 (34.4%)	<.001	67 (2.2%)
Obesity (body mass index >30 kg/m²)	1050 (35.4%)	743 (33.3%)	307 (41.5%)	<.001	65 (2.1%)
Diabetes	1307 (43.2%)	876 (38.6%)	431 (57.0%)	<.001	7 (0.2%)
Hypertension	2747 (90.8%)	2042 (90.0%)	705 (93.0%)	.017	7 (0.2%)
Smoking status					
Current smoker	358 (11.9%)	271 (12.0%)	87 (11.5%)	<.001	23 (0.8%)
Non-smoker	1242 (41.3%)	999 (44.3%)	243 (32.2%)		
Former smoker	1410 (46.8%)	985 (43.7%)	425 (56.3%)		
Cirrhosis	50 (1.7%)	40 (1.9%)	10 (1.4%)	.495	174 (5.7%)
Respiratory disease	708 (23.9%)	463 (20.9%)	245 (32.7%)	<.001	68 (2.2%)
Cognitive disorder	19 (0.6%)	10 (0.5%)	9 (1.2%)	.034	78 (2.6%)
Cancer	621 (21.2%)	478 (21.8%)	143 (19.4%)	.193	103 (3.4%)
Dyslipidemia	2223 (73.6%)	1558 (68.9%)	665 (87.7%)	<.001	14 (0.5%)
Low birth weight (<2500 g)	215 (9.8%)	158 (9.7%)	57 (10.4%)	.687	846 (27.9%)
**Laboratory data (at baseline)**					
eGFR (ml/min/1.73 m²)	32.8 [23.6,43.1]	33.3 [23.9,43.5]	30.9 [22.9,41.4]	.001	.
eGFR slope (ml/min/1.73 m²/year)	−1.53 [−5.35,2.02]	−1.54 [−5.32,1.81]	−1.50 [−5.43,2.42]	.31	.
Urinary albumin-creatinine ratio category					
Normal or low (A1)	834 (30.2%)	639 (30.8%)	195 (28.3%)		
Moderately increased (A2)	969 (35.0%)	718 (34.6%)	251 (36.5%)	.453	268 (8.8%)
Severely increased (A3)	962 (34.8%)	720 (34.7%)	242 (35.2%)		
Sodium (mmol/l)	140 [139 142]	140 [139 142]	140 [139 142]	.593	16 (0.5%)
Potassium (mmol/l)	4.50 [4.20,4.90]	4.50 [4.20,4.90]	4.50 [4.20,4.90]	.897	13 (0.4%)
Chlorine (mmol/l)	104 [102 107]	104 [102 107]	103 [101 106]	<.001	195 (6.4%)
Uric acid (µmol/l)	426 [348 505]	416 [345 494]	452 [369 541]	<.001	250 (8.2%)
HbA1c (%)	6.00 [5.50,7.00]	5.90 [5.50,6.80]	6.30 [5.72,7.30]	<.001	600 (19.8%)
Hemoglobin (g/dl)	12.9 [11.8,14.1]	13.0 [11.8,14.1]	12.9 [11.8,14.1]	0.454	23 (0.8%)
Albumin (g/l)	40.3 [38.0,43.0]	40.7 [38.0,43.0]	40.0 [37.6,42.0]	.003	489 (16.1%)
Ferritin (ng/ml)	128 [71.0 228]	134 [75.0 233]	113 [62.2 220]	.001	459 (15.1%)
CRP (mg/l)	3.90 [1.80,7.80]	3.40 [1.60,7.00]	4.40 [2.20,10.0]	<.001	1473 (48.6%)
Total cholesterol (g/l)	4.71 [3.96,5.61]	4.87 [4.11,5.75]	4.25 [3.62,5.15]	<.001	334 (11.0%)
HDL (g/l)	1.24 [1.01,1.53]	1.26 [1.03,1.60]	1.16 [0.948,1.42]	<.001	355 (11.7%)
LDL (g/l)	2.56 [1.90,3.35]	2.69 [2.01,3.45]	2.23 [1.70,2.92]	<.001	414 (13.6%)
Triglycerides (g/l)	1.55 [1.11,2.25]	1.52 [1.10,2.25]	1.60 [1.15,2.28]	.036	343 (11.3%)
**Cardiac ultrasound**					
Available cardiac ultrasound data	1254 (48.4%)	815 (42.0%)	439 (67.6%)	<.001	444 (14.6%)
Left ventricular ejection fraction (%)	61.0 [55.0,68.0]	65.0 [60.0,70.0]	58.5 [45.0,65.0]	<.001	301 (24.0%)
Left ventricular hypertrophy	234 (18.7%)	132 (16.2%)	102 (23.2%)	.001	421 (33.6%)
Drug prescriptions at baseline					
Diuretics	1650 (54.6%)	1106 (48.8%)	544 (71.8%)	<.001	9 (0.3%)
RAAS inhibitors	2294 (75.9%)	1739 (76.7%)	555 (73.2%)	.056	9 (0.3%)
Beta-blockers	1269 (41.8%)	779 (34.2%)	490 (64.6%)	<.001	9 (0.3%)
Lipid-lowering agents	1908 (63.1%)	1312 (57.9%)	596 (78.6%)	<.001	9 (0.3%)
Proton pump inhibitors	991 (32.8%)	638 (28.2%)	353 (46.6%)	<.001	9 (0.3%)
Antigout drugs	1025 (33.9%)	740 (32.7%)	285 (37.6%)	.015	9 (0.3%)
Anti-inflammatory	43 (1.4%)	38 (1.7%)	5 (0.7%)	.061	9 (0.3%)
Polypharmacy	1006 (33.3%)	615 (27.1%)	391 (51.6%)	<.001	9 (0.3%)
Adherence to medication					
Good	1129 (37.6%)	853 (37.9%)	276 (36.6%)	.801	31 (1.0%)
Moderate	1651 (55.0%)	1229 (54.7%)	422 (56.0%)		
Poor	222 (7.4%)	166 (7.4%)	56 (7.4%)		

Quantitative values are presented by median [Q1, Q3], qualitative values are presented by frequency and percentage

### Incidence of AKI and MACE

Over a median follow-up of 5.2 years [3.2–5.9], a total of 530 patients experienced at least one episode of AKI in a median delay of 1.7 years. These events were predominantly of mild severity (71.7% stage 1), of pre-renal etiology (72.5%), and required hospitalization (78.1%). Concurrently, there were 498 major cardiovascular events, 628 KFRT, and 316 non-cardiovascular deaths.

**Figure 1:**
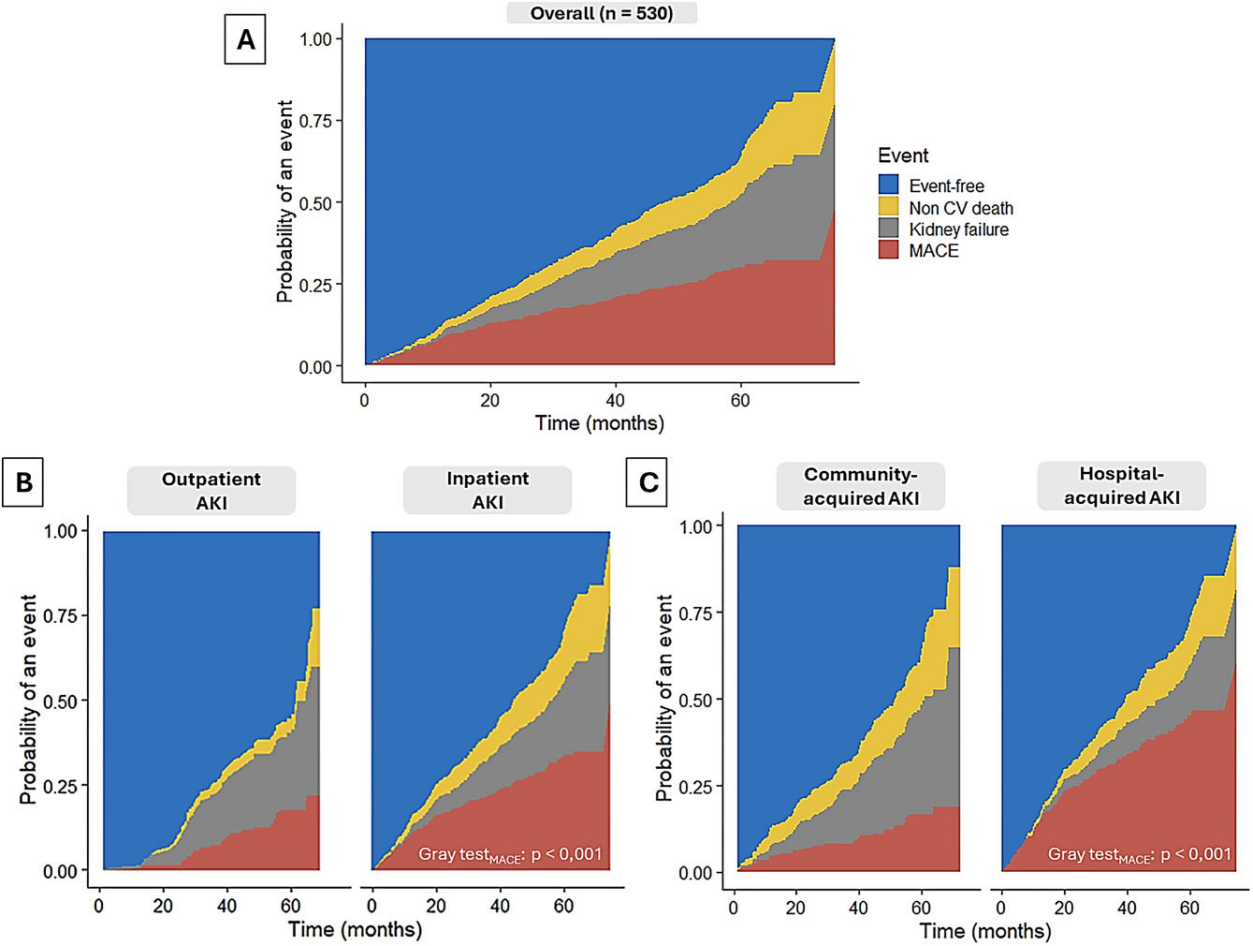
Cumulative incidences of MACE, kidney failure, and non-cardiovascular death after a first incident AKI episode during follow-up (*N* = 530): (**A**) overall, (**B**) according to out-/inpatient status of the AKI episode, and (**C**) according to community-/hospital-acquired status of the AKI episode.

Following an AKI episode, the estimated cumulative incidences of MACE, KFRT, and non-cardiovascular death were 8.1% (6.1–10.8), 1.3% (0.6–2.8), and 2.3% (1.3–4.0), respectively, at 12 months; 18.5% (15.5–22.1), 11.5% (9.1–14.6), and 6.0 (4.3–8.4), respectively, at 36 months; and 30.1% (26.4–34.3), 22.1% (18.8–26.0), and 11.2% (8.8–14.3), respectively, at 60 months (Fig. 1A). The cumulative incidence of MACE after an AKI episode appeared significantly higher for inpatient (vs outpatient) and hospital-acquired (vs community-acquired) events (*P* < .001) (Fig. 1B and C). In total, subsequent MACE occurred at a median of 6.7 months (1.8–16.6) after the AKI episode.

### Association between incident AKI events and subsequent MACE

**Figure 2:**
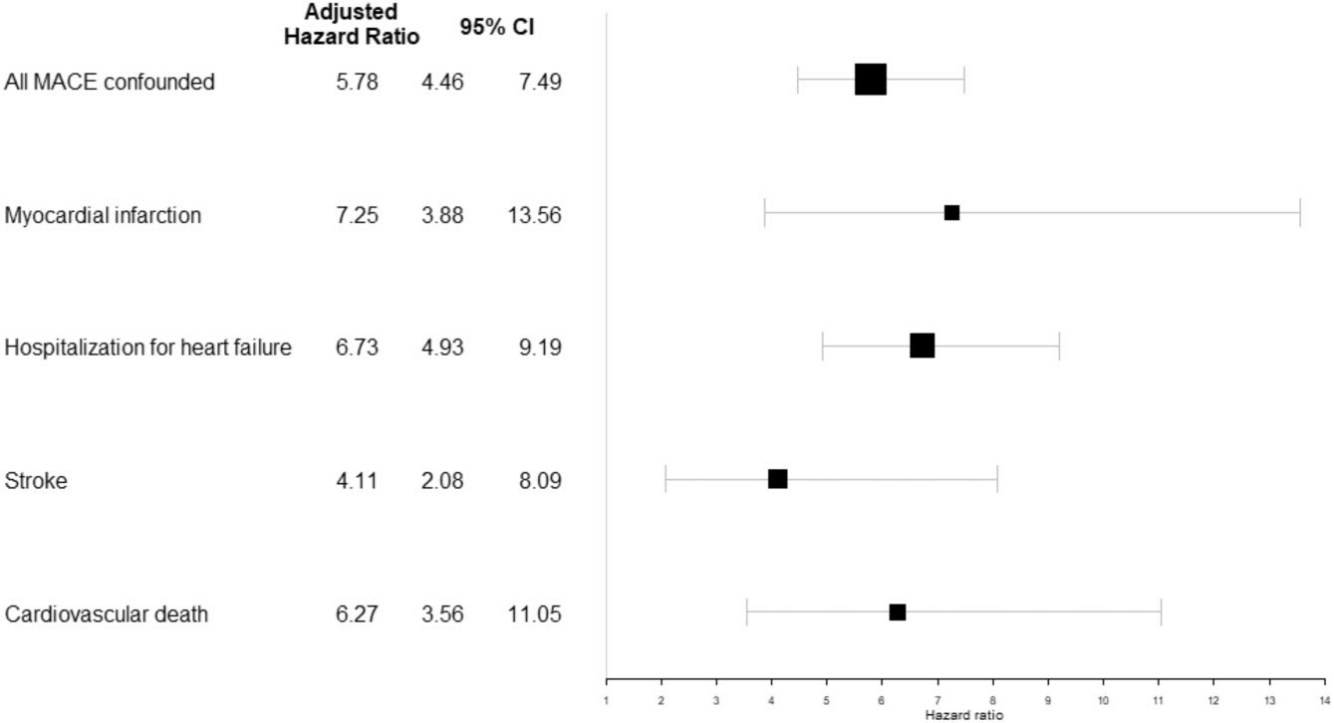
Adjusted hazard ratios (95% confidence interval) of subsequent MACE associated with incident AKI event within the entire cohort (*N* = 3033, number of AKI events = 530, number of MACE = 498).

In the entire cohort, the adjusted HR (95%CI) for subsequent MACE was more than five times higher in patients with, versus without, an AKI event during follow-up (HR: 5.78 (4.46–7.49), *P* < .001) (Fig. 2).

**Figure 3:**
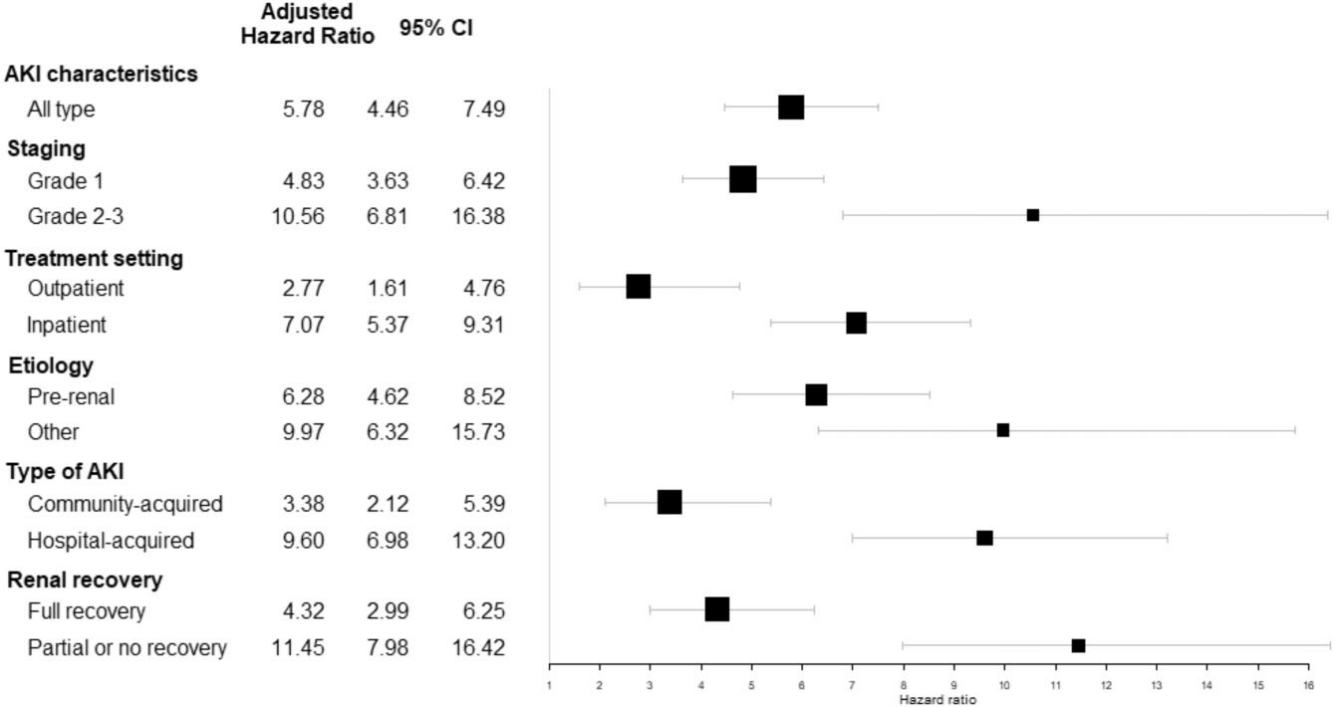
Adjusted hazard ratios (95% confidence interval) of subsequent MACE associated with incident AKI event within the entire cohort (*N* = 3033), stratified by AKI characteristics.

This association was also confirmed for each of the different events defining MACE, with a similar magnitude for acute myocardial infarction, hospitalization for heart failure, and cardiovascular death. It appeared slightly less pronounced concerning strokes (HR: 4.11 (2.08–8.09), *P* < .001) (Fig. 2). The excess risk of subsequent MACE appeared higher for stage 2–3 AKI (vs stage 1), those requiring or complicating hospitalization (vs outpatient or community-acquired), and those for which complete renal recovery was not observed on hospital discharge (Fig. 3).

Overall, the fraction of MACE risk attributable to AKI episodes within this population was estimated at 18.1% (13.6–22.4).

### Interaction and sensitivity analyses

**Figure 4:**
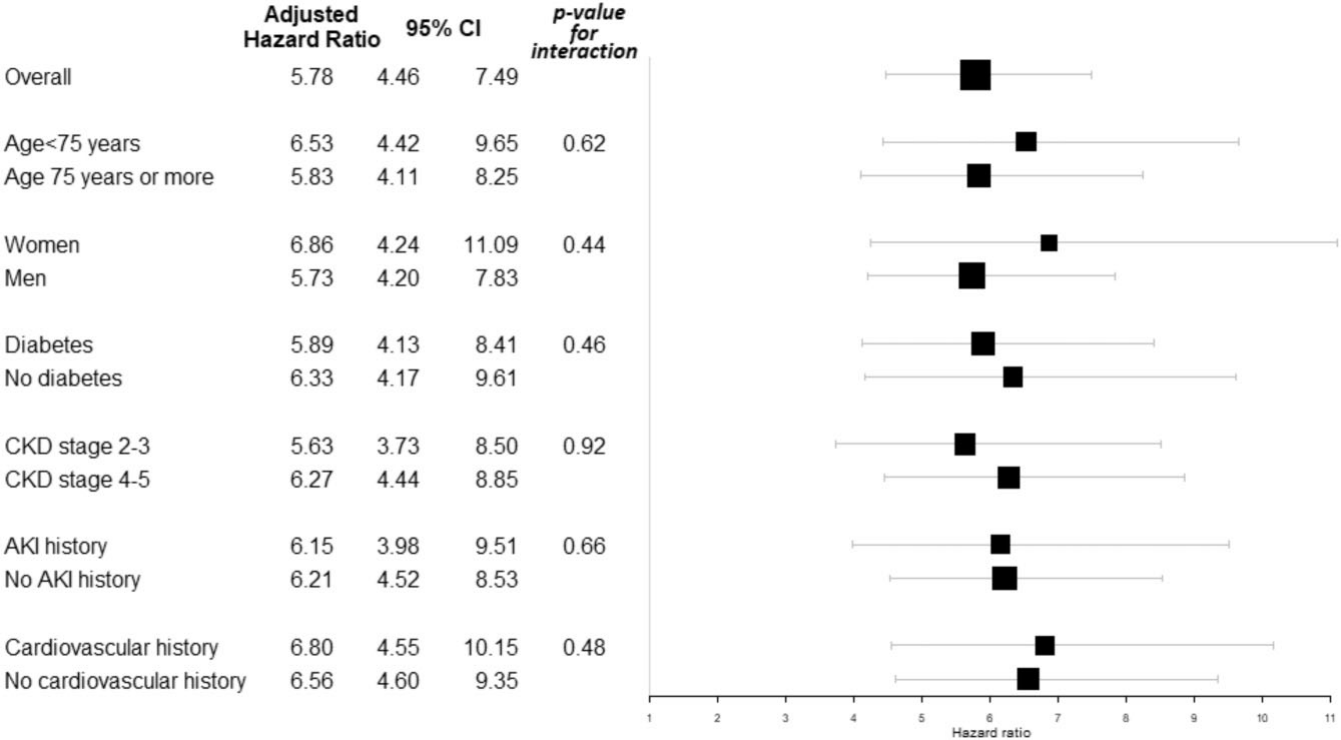
Interaction analyses: adjusted hazard ratios (95% confidence interval) of subsequent MACE associated with incident AKI event within the entire cohort (*N* = 3033), according to different subgroups of patients.

No interaction with age, sex, history of diabetes, CKD stage, AKI, or cardiovascular event was observed; the association between incident AKI and subsequent MACE was significant across all subgroups with a comparable effect size (Fig. 4). Although still highly significant, the associations were less pronounced when concurrent AKI–MACE events were censored (Supplementary Table S1) and globally similar when patients with a previous history of AKI and/or cardiovascular events before inclusion were excluded (Supplementary Table S2).

## DISCUSSION

This study identifies AKI as an independent risk factor for MACE within the CKD patient population, attributing to it an estimated contribution of nearly 20%. It encompasses all types of MACE, with a 5-fold increase in the risk of subsequent hospitalization due to heart failure, acute myocardial infarction, or cardiovascular death following an AKI episode in this population. The elevated risk of stroke, although notable, is less pronounced by comparison. On average, MACE manifests ∼6 months post-AKI, underscoring the critical need for vigilant monitoring following such an event.

While the heightened risk of mortality in both the short and long term following AKI is well-documented, the potential mediation of this risk through increased cardiovascular vulnerability remains uncertain [12, 31]. Currently, data rigorously examining the connection between AKI and the subsequent risk of MACE is sparse, predominantly originating from studies involving highly specific populations such as those undergoing cardiac surgery, coronary revascularization, experiencing contrast-induced AKI, or patients in intensive care units [32–34, 35]. A meta-analysis by Odutayo *et al.*, which encompassed >250 000 patients from 25 studies, primarily reflecting populations with significant cardiovascular risks, acknowledged an association between AKI and an elevated risk of MACE. However, it also pointed out considerable limitations, including substantial heterogeneity in exposure definitions and follow-up durations. [13] Conversely, research by Go *et al.*, based on nearly 150 000 hospitalized patients within the Kaiser Permanente Northern California system with or without AKI, identified an increased risk of heart failure only within the first year of follow-up [12]. The discrepancies observed in comparison to our study might be attributable to a shorter follow-up period and the relatively preserved kidney function in most patients. In recent findings from a study of 2177 adults in the Chronic Renal Insufficiency Cohort, McCoy *et al.* revealed a correlation between AKI episodes and the risk of heart failure and atherosclerotic cardiovascular events over an average follow-up time of 3 years [11]. When juxtaposed with existing literature, the effect sizes noted in our study are particularly pronounced, potentially indicative of an additive impact of CKD and AKI, where the combined influence of each may manifest in a multiplicative manner [7, 36].

A significant strength of our investigation is the detailed collection, verification, and granularity of event-related information throughout patient follow-up [18, 37]. Each AKI episode underwent rigorous validation by a panel of experts, enabling the inclusion of specific details on severity, context, and the adjudicated etiology in our analyses [22]. This research thus reaffirms the potentially detrimental effect of even moderately severe AKI episodes (i.e. grade 1), including those not necessitating hospitalization or followed by rapid renal recovery. As previously established, even modest increases in serum creatinine levels should be regarded as non-trivial events [11, 38, 39]. Moreover, we observed a severity–response correlation, with a stronger association between the severity markers of AKI episodes and subsequent MACE risk, aligning with previous literature findings [12, 14]. Another distinctive aspect of this study is the consideration of often overlooked confounding factors; unlike prior studies, we accounted for potential withdrawals of ACE inhibitors or ARBs following AKI episodes when estimating MACE risk. Similarly, in alignment with McCoy *et al.*’s recent study, our analyses adjusted for eGFR level and slope, acknowledging their potential influence on the association between AKI and subsequent MACE risk [11].

Our findings call for further exploration of what we describe as the “butterfly effect” of AKI episodes on the cardiovascular system [7]. This concept pertains to the acute alterations occurring during an AKI episode that initiate pathophysiological responses within the cardiac muscle, potentially culminating in cardiovascular events over time. Previous research has demonstrated that the early and substantial release of interleukin-33 (IL-33) following ischemic-reperfusion AKI is associated with long-term cardiac hypertrophy and dysfunction [40]. Interestingly, animal models have shown that inhibiting IL-33 release during AKI episodes can protect against cardiac remodeling, suggesting a viable therapeutic approach for CKD patients. Another study highlighted the significance of Galectin-3, noting its specific upregulation post-AKI as a precursor to enduring cardiac remodeling in a mouse model of renal ischemia-reperfusion, warranting further investigation [41]. Recent research on IL-11 further highlights its role in inducing heart failure, hypertrophy, and fibrosis. Studies in mouse models have shown that anti-IL-11 treatments can prevent renal fibrosis in both AKI and CKD, which may represent a novel therapeutic avenue. Given the emerging relevance of IL-11, as discussed by Widjaja *et al.* [42], its potential impact on cardiovascular and renal outcomes should be further explored in future studies. Therefore, it is critical to consider targeting these pathways during the course of AKI or introducing cardioprotective drugs such as iSGLT2 inhibitors and ACE inhibitors in the immediate aftermath of AKI. The latter is now being evaluated in an ongoing trial (NCT05272878). It is also important to acknowledge that SGLT-2 inhibitors and GLP-1 analogs, which were not included in our analysis due to the cohort being established before the completion of relevant RCTs, could potentially influence the incidence of AKI and its association with MACE. While it is plausible that the inclusion of these medications might only numerically alter our findings without affecting the overall conclusions, this remains speculative. Future studies should consider the potential impact of these therapies on the AKI–MACE relationship to ensure a more comprehensive understanding. While the connection between AKI and stroke risk is less emphasized, as corroborated by prior studies, recent research has unveiled several potential pathophysiological mechanisms, including disruptions to the blood–brain barrier, systemic inflammation/neuroinflammation, and impaired coagulation and thrombosis balance [43].

Our research findings compellingly indicate that episodes of AKI, including those not requiring hospitalization or deemed less severe, are associated with substantial increased risk of cardiovascular complications in patients with CKD. This situation demands a 2-fold approach in patient management. First, there is a critical need for the prevention of AKI episodes, underscored by the importance of enhanced patient education regarding their medication regimen, including diuretics, renin-angiotensin system blockers, and mineralocorticoid receptor antagonists. This recommendation is particularly relevant given the proportion of pre-renal causes of AKI identified in our study, coupled with the high incidence of severe adverse drug reactions in this population [44, 45]. Moreover, there is an urgent need for improved communication among various healthcare providers, acknowledging that AKI episodes are frequently underreported in medical records, especially among CKD patients, where such incidents may be minimized [22, 46]. Special emphasis should be placed on meticulous monitoring of these patients following an AKI episode, considering the significantly elevated risk of cardiovascular incidents, even in the short term [47, 48]. Previous research demonstrated that the initial consultation with the referring nephrologist post-AKI could be delayed by nearly 3 months [22]. Based on our findings, healthcare providers should consider implementing: (i) early post-AKI consultations to monitor renal recovery and assess cardiovascular risk; (ii) regular cardiovascular assessments to detect early signs of potential complications; (iii) enhanced patient education regarding the importance of medication adherence and lifestyle modifications to reduce cardiovascular risk; and (iv) consideration of cardioprotective therapies, such as iSGLT2 inhibitors and ACE inhibitors, in the immediate aftermath of AKI. These measures can help mitigate the heightened cardiovascular risk observed in CKD patients following an AKI episode, potentially improving long-term outcomes.

Nonetheless, our study acknowledges several limitations. The observational nature of this study precludes definitive conclusions about the causality of the observed relationships, especially because the identification of events relied on medical records and patient interviews, which could lead to misclassification. While our data indicate that a portion of the cohort had a reduced ejection fraction (left ventricle ejection fraction <45%) in the subgroup with a history of AKI at baseline, it is important to acknowledge that heart failure with preserved ejection fraction is more commonly associated with CKD. Given that heart failure with preserved ejection fraction has only recently been clearly defined as a distinct clinical entity, it is likely that its incidence in our cohort was similar, even if not formally identified. This limitation should be considered when interpreting the cardiovascular outcomes in this study. However, the prospective design, along with the careful identification and classification of AKI and MACE events, lends substantial validity to our investigation. While cohort studies inherently face the challenges of attrition bias and residual confounding, the low lost to follow-up rate during active monitoring (<5%) and the triangulation of data from various sources, including national registries, stand as strengths of our research. The *a priori* selection of potential confounders using a directed acyclic graph and the robustness of the observed association minimized the risk for confounding bias. Although our study accurately reflects nephrological care in France, the inclusion of patients under nephrological follow-up may introduce a selection bias. Nevertheless, our findings imply that the association could be underestimated for patients with less frequent medical follow-up. A limitation could stem from the analysis being confined to the first AKI episode during the follow-up period, not considering the potential prognostic significance of recurrent events. However, further analyses revealed no interaction with a history of past-AKI, suggesting that the adverse impact of AKI is predominantly not influenced by its recurrence. Another limitation is that the diagnosis of AKI is made through laboratory workup, and patients may be asymptomatic. This could lead to an underestimation of the number of patients who experienced AKI, particularly in cases of community-acquired AKI. Another limitation of our study is the potential misclassification of some stage 1 AKI cases, particularly among outpatients who achieve full recovery without hospitalization. It is possible that these cases reflect a response to ACE inhibitor/ARB/MRA overdosing or up-titration, especially given the expected presence of renal artery stenosis or diabetic nephropathy in this cohort. As with our earlier CKD-REIN papers, our approach does not allow for precise differentiation in such instances.

In summary, our study highlights a pronounced severity–response relationship between the severity of AKI events and the increased risk of subsequent MACE in a cohort of CKD patients. These results propose that AKI serves not only as an indicator of increased cardiovascular risk but may also play a role as a mediator between CKD and this risk. Heightened awareness within the medical community, coupled with the adoption of preventive strategies and therapeutic interventions, holds the potential to significantly enhance patient outcomes.

## Supplementary Material

sfae337_Supplemental_File

## Data Availability

Data set is available upon request to the CKD-REIN Consortium. As data are not available on an open science platform, access to the data should be requested to Dr Natalia Alencar de Pinho (CKD-REIN cohort coordinator).
