# Supplementary material for: Acute kidney injury as a key predictor of cardiovascular events in chronic kidney disease patients: the CKD-REIN study
Source: Clin Kidney J. 2024 Dec 11;17(12):sfae337. doi: 10.1093/ckj/sfae337 (PMC11646099; doi:10.1093/ckj/sfae337)
Supplement: sfae337_Supplemental_File [file sfae337_supplemental_file.pdf]

Supplementary material

Acute Kidney Injury as a Key Predictor of Cardiovascular Events in Chronic Kidney Disease Patients: the CKD-REIN study

Nans Florens, Estelle Aymes, Victoria Gauthier, Luc Frimat, Maurice Laville, Dimitri Bedo, Thomas Beaudrey, Philippe Amouyel, Nicolas Mansencal, Céline Lange, Sophie Liabeuf, Ziad A. Massy, Benedicte Stengel, Natalia Alencar de Pinho, Aghiles Hamroun

Table S1: Sensitivity analysis: Adjusted Hazard Ratios of subsequent MACE associated with incident AKI event, after censoring of concomitant AKI-MACE events

Table S2: Sensitivity analysis: Adjusted Hazard Ratios of subsequent MACE associated with incident AKI event, after exclusion of patients with a history of AKI or cardiovascular event at baseline

Table S1. Sensitivity analysis: Adjusted Hazard Ratios (95% Confidence Interval) of subsequent MACE associated with incident AKI event, after censoring of concomitant AKI-MACE events (N = 3,033, number of AKI events = 444).

Outcome	Adjusted Hazard ratio (95% confidence interval)	*p-value*
**All MACE confounded**	2.69 (2.04 – 3.55)	< 0.001
**Myocardial infarction**	5.50 (2.93 – 10.30)	< 0.001
**Hospitalization for heart failure**	1.97 (1.38 – 2.79)	< 0.001
**Stroke**	3.49 (1.75 – 6.96)	<0.001
**Cardiovascular death**	3.58 (1.94 – 6.63)	< 0.001

Table S2. Sensitivity analysis: Adjusted Hazard Ratios (95% Confidence Interval) of subsequent MACE associated with incident AKI event, after exclusion of patients with a history of AKI or cardiovascular event at baseline (N = 1,820, number of AKI events = 245, number of MACE = 188).

Outcome	Adjusted Hazard ratio (95% confidence interval)	*p-value*
**All MACE confounded**	6.69 (4.28 – 10.46)	< 0.001
**Myocardial infarction**	6.43 (2.47 – 16.73)	< 0.001
**Hospitalization for heart failure**	9.59 (5.24 – 17.55)	< 0.001
**Stroke**	4.90 (1.71-14.04)	<0.001
**Cardiovascular death**	6.22 (2.34 – 16.54)	< 0.001
